# A Novelty System for Biotization of Plant Microshoots and Collection of Natural Compounds

**DOI:** 10.3390/mps2010005

**Published:** 2019-01-07

**Authors:** Mário Rui da Costa Basílio e Castro, Carla Ragonezi, Paulo Guilherme Leandro de Oliveira, Maria Amely Zavattieri

**Affiliations:** 1 Institute of Mediterranean Agricultural and Environmental Sciences (ICAAM). Colégio da Mitra, Ap. 94, 7002-554 Évora, Portugal; mrcbcastro@gmail.com; 2 Banco de GermoplasmaISOPlexis, Campus da Penteada, Universidade da Madeira, 9020-105 Funchal, Portugal; zavattieri@uevora.pt; 3 Departamento de Biologia, Pólo da Mitra Apartado 94, 7002-554 Évora, Portugal; oliveira@uevora.pt; 4 Research Centre in Biodiversity and Genetic Resources Évora (CIBIO-UE), Ap. 94, 7002-554 Évora, Portugal; 5 Instituto de Ciências da Terra (ICT), Colégio Luís António Verney, Rua Romão Ramalho 59, 7000-671 Évora, Portugal

**Keywords:** Keywords: biotechnology, biotization, chemical analysis, double-phase medium, phenolic compounds, liquid chromatography—diode array detector—mass spectrometry

## Abstract

An in vitro plant microshoot culture system composed of two phases; a liquid phase overlaid by a floating solid phase, which is described in detail herein. This system is designed to enable the extraction of natural compounds released/disseminated into the liquid phase during root growth, thus facilitating their processing and biochemical characterization. The solid phase holds the plant afloat and enables the simultaneous culture of a microorganism, yet avoiding its penetration into the liquid phase, where the roots are submerged. Both phases can be independently formulated as required for growth optimization of both organisms. Considering the closed system and known variables described in this patent, applications of the described method include testing with pesticides, herbicides, and other similar products.

## 1. Introduction

Many crucial root functions involve interactions with symbionts or rhizosphere-dwelling microorganisms. This often demands the design of biotization experiments that adequately model such interdependencies [1]. Our efforts to biochemically characterize the compounds that result from the interaction between symbiont fungi and cloned stone pine microshoots were met initially with enormous difficulties, due to interference from the gelling media utilized. Thus, we proceeded to develop a two-phase biotization system that allows the collection of root exudates in an underlying liquid medium, free from gelling agent interference and free from direct contact with growing microorganism [2,3]. The new system was patented under the Portuguese national patent number PT 105239 [3], and the present patent summary makes it available to the wider scientific community.

## 2. Materials and Methods

### 2.1. Preparation of the Culture System

Materials needed: Culture media, one for the solid phase and the other for the liquid phase; inert floater; sterile transparent containers with caps; a sterile curved spatula; pipettes and 5/10 mL tips.

### 2.2. Procedures

This is a general procedure that can be adjusted according to experimenter needs. In the Results section, one example of implementation is provided.

**Figure 1:**
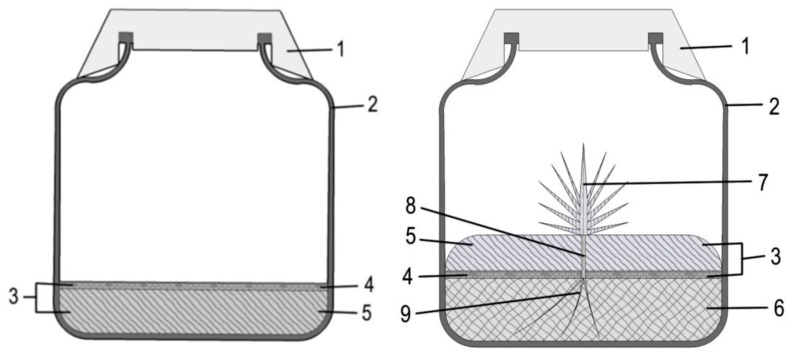
Illustrative scheme of the double-phase culture media system for in vitro biotization between plants and microorganisms. The finished system is shown on the right, and the initial step of gelling the solid phase before inverting is shown on the left. 1, cover; 2, flask; 3, solid phase (4 + 5); 4, floater substrate; 5, gelled culture medium; 6, liquid phase; 7, shoot; 8, stem; 9, roots.

The numbers in brackets refer to Figure 1. Notes are added at the end of the procedure.

- The solid culture medium (5) and the liquid culture medium (6) are aseptically prepared—note 1;
- each flask is floored with the inert floater (4)—note 2;
- the medium supplemented with gelling agent is poured into the flasks. The inert floater will reach the surface before gelling of the medium (Figure 1, left);
- after gelling, the solid medium must be inverted; this is achieved by inserting the curved spatula between the solid medium and the inner flask surface, and carefully lifting it and turning it upside down, so that the inert floater faces downward;
- the curved spatula is then used to move the edge of the solid phase, creating an opening that allows pipet insertion of the liquid phase underneath the solid phase—note 3;
- the floating solid phase is pierced for insertion of the plant(s), by making an opening in the upper solid medium using a heat sterilized perforating tool (e.g., wide tweezers)—note 4;
- each plant is inserted in the hole that was made in the solid phase, such that the root system (9) is immersed into the liquid phase—note 5. At this point, the essential part of the system is set (Figure 1, right).
- Optionally, other organisms are placed on the solid phase, at the interface with the air compartment.
- As the culture proceeds, samples from the liquid phase can be collected for biochemical analysis, by inserting a pipette between the solid phase and the flask inner surface.

### 2.3. Notes

1. In step a, culture media formulation may vary depending on the species and culture purpose including the possibility of the same medium being used for both phases, except for the absence of carbohydrates in the liquid phase;
2. in step b, the amount of inert floater depends on the size of the flask but should create a layer with approximately 4 to 5 mm thickness for stability purposes;
3. in step e, use for pipetting a 5 or 10 mL tip;
4. alternatively, step f can be done between steps d and e;
5. in step g, careful operation is required to avoid damaging the root system.

## 3. Results

The system (Figure 1) was devised for our biotization experiments with rooted *Pinus pinea* microshoots [2,3,4], which will serve as guideline for other implementations.

A convenient implementation consisted of 100 mL glass flasks with plastic caps, such as the Magenta^®^ B-cap from Sigma-Aldrich (St. Louis, MO, USA), containing a solid phase made of 15 mL agar culture medium with the pH adjusted to 5.8 before autoclaving for 20 min at 121 °C, together with 0.9 g of expanded perlite (horticultural grade, 3.36–4 mm) serving as the inert floater, and a liquid phase of 30 mL liquid culture medium. For most experiments, one rooted pine microshoot of approximately 4 cm shoot height was used per flask, but up to three microshoots can be set up in these flasks. Plant cultures were carried at a 16 h photoperiod, 24 °C/19 °C day/night, respectively, and after 7 days of adaptation, fragments of an ectomycorrhizal fungus pure culture were introduced into the system. All experiments included control flasks without biotization. Liquid phase sampling (5 mL) was done after as many as five time-points, and, during the experiment, no signs of fungus penetration of the plant, or dissemination to the liquid phase, was observed. Liquid medium was added when necessary. Plant mortality was negligible, and plant growth in inoculated flasks was unaltered by comparison with controls. Root growth proceeded normally, and the plants were suitable for downstream procedures for out-planting.

After our experiments of stone pine biotization with *Pisolithus arhizus* (Scop.) Rauschert using this implementation, the collected liquid phase samples were analyzed with high performance liquid chromatography (HPLC-UV) and liquid chromatography—diode array detector—mass spectrometry (LC-DAD-MS), resulting in the isolation and identification of an ester of *o*-coumaric acid [4]. This phenolic compound was not cited before as a signaling substance between a pine root and an ectomycorrhizal fungus.

## 4. Discussion

The patented double-phase medium system is perfectly suitable as a biotization design, supporting the optimal growth of both host and symbiont organisms, and additionally enabling the analysis of chemical mediators released by the roots into the lower liquid phase. Considering that proper root growth is a key factor for further strengthening and establishment of the plants during all phases of acclimation [5], determining the biochemical substances responsible for better plant performance may lead to the production of those substances by artificial synthesis, foreseeing posterior application in large scale culture designs. The results obtained with this system contributed to the characterization of a novel biochemical signal involving conifer species and an ectomycorrhizal fungus, clearly demonstrating the potential of the patented system, as it was developed, to quite effectively study a great variety of plant-microorganism combinations, specifically when the objective is to identify released natural compounds.

## 5. Patents

The system was patented under the Portuguese national patent number PT 105239 [3].
